# Practices, knowledge, and concerns for out-of-home firearm storage among those with access to firearms: results from a survey in two states

**DOI:** 10.1186/s40621-023-00426-9

**Published:** 2023-03-13

**Authors:** Leslie M. Barnard, Rachel L. Johnson, Sara Brandspigel, Lauren A. Rooney, Megan McCarthy, Frederick P. Rivara, Ali Rowhani-Rahbar, Christopher E. Knoepke, Ryan A. Peterson, Marian E. Betz

**Affiliations:** 1 Department of Epidemiology, Department of Emergency Medicine, Colorado School of Public Health, University of Colorado School of Medicine, Aurora, CO USA; 2 Department of Emergency Medicine, School of Medicine, University of Colorado Anschutz Medical Campus, Aurora, CO USA; 3 Department of Biostatistics and Informatics, Colorado School of Public Health, University of Colorado Anschutz Medical Campus, Aurora, CO USA; 4 Injury and Violence Prevention Center, Colorado School of Public Health, University of Colorado Anschutz Medical Campus, Aurora, CO USA; 5 Firearm Injury Policy and Research Program, Harborview Injury Prevention and Research Center, University of Washington, Seattle, WA USA; 6 Department of Epidemiology, School of Public Health, University of Washington, Seattle, WA USA; 7 Division of Cardiology, School of Medicine, University of Colorado Anschutz Medical Campus, Aurora, CO USA; 8 Adult and Child Consortium for Outcomes Research and Delivery Science, School of Medicine, University of Colorado Anschutz Medical Campus, Aurora, CO USA

**Keywords:** Suicide prevention, Firearm storage, Epidemiology

## Abstract

**Background:**

Temporary, voluntary storage of firearms away from the home is a recommended option for individuals with risk of suicide, but it may also be used in other situations (e.g., long trips). Prior work has explored the availability of storage options and the views of storage locations. Little is known about out-of-home storage practices among those who live in homes with firearms (including owners).

**Methods:**

We surveyed English-speaking adults (18 or older) in two states (Colorado and Washington) living in a home with a firearm (June–July 2021).

**Results:**

Among the final sample of 1029, most respondents were white (88.1%) and non-Hispanic (85.0%); half were female (50.8%), and the most common age group was ages 35–44 (25.5%). Just over one quarter (27.3%) of respondents indicated they had stored a firearm away from their home/car/garage in the last 5 years. The place most respondents said they were somewhat or very likely to consider was at a family members home (62.7%) or at a self-storage facility (52.5%).

**Conclusion:**

Out-of-home firearm storage is a relatively common practice and endorsed by many gun-owners, suggesting out-of-home storage is feasible for firearm owners as an approach to suicide prevention.

**Supplementary Information:**

The online version contains supplementary material available at 10.1186/s40621-023-00426-9.

## Background

Voluntary out-of-home firearm storage is a safety-promotion practice used by firearm owners in a variety of different circumstances including when a visitor to the home is prohibited from firearm possession, grandkids are visiting, homeowners are taking an extended trip, military deployment, when owners are renting or selling a home, or when someone in the home is at risk of suicide. Lethal means safety programs seek to limit access to firearms during a time of crisis, putting space between the person who is at risk and highly-lethal means until a crisis period has passed, thereby preventing suicide (Allchin et al. 2019; Barber and Miller 2014).Out-of-home storage of personal firearms is a component of lethal means safety and is recommended by many health professional and firearm-related organizations for those at risk of suicide (Allchin et al. 2019; Barber and Miller 2014). Currently, it’s unclear how temporary, voluntary out-of-home firearm storage is practiced and for what reasons or circumstances firearms are being stored outside of the home.

In an effort to help firearm owners identify legal storage locations outside the home, public health professionals in Colorado developed the first statewide map showing firearm ranges, retailers, and law enforcement agencies willing to consider requests for voluntary firearm storage (Kelly et al. 2020). Subsequently, online maps of storage locations have been developed in other states including Washington, Maryland, North Carolina, New Jersey, and New York (Washington Firearm Safe Storage Map—Harborview Injury Prevention and Research Center 2022; Bongiorno et al. 2021; NJ Firearm Storage Map|New Jersey Gun Violence Research Center 2022; New York Firearm Storage Map. Rockefeller Institute of Government 2022; Map and Marylanders to Prevent Gun Violence 2022). However, the awareness of and acceptability of these maps from the perspective of individuals with firearms in their homes has not been evaluated.

To our knowledge, this is the first survey to examine the practices of those who live in homes with firearms (including owners) regarding out-of-home firearm storage. We sought to understand (1) how often out-of-home firearm storage is used, (2) where people are storing their firearms when they do so outside of the home, (3) the circumstances surrounding when people choose to store outside of the home, (4) how people would use out-of-home storage hypothetically in the future; and (5) if those with access to firearms know about the firearm storage maps and (6) their perception of barriers or facilitators to using firearm storage maps.

## Methods

### Survey instrument & implementation

A survey instrument was developed based on the Exploration, Preparation, Implementation, Sustainment (EPIS) framework (Framework 2022) to examine prior or possible future out-of-home firearm storage, perceived barriers and facilitators to use of firearm storage maps, policy recommendations, and optimal avenues for public education about out-of-home storage. The EPIS framework identifies key factors and interactions within them to facilitate and sustain implementation (Moullin et al. 2019). The survey instrument was pre-tested with individuals knowledgeable about firearms and survey research, including a firearm retailer, member of law enforcement, and several researchers external to the project to determine question clarity and appropriateness of response options. The final survey was 41 items and took less than 10 min to complete (see Additional file 1: Appendix for full survey). Eligible participants were English-speaking adult (18 or older) residing in either Colorado or Washington state in a home with a firearm (either as the firearm owner or not). We included family members because they may be the ones requesting temporary storage and are therefore key users. The survey was administered by Qualtrics through sampling of existing managed panels to reach a diverse sample. Quality control checks to avoid duplicates include digital fingerprinting technology and IP address checks. All survey recruitment and disbursement of incentives occurred via Qualtrics. Quota-based sampling was employed to ensure that at least 50% of the respondents were from men. The survey was implemented in June and July of 2021. This study was deemed exempt by the Colorado Multiple Institutional Review Board (IRB) and the University of Washington IRB.

### Analysis

Qualtrics monitored survey data and completed data quality checks, including replacing respondents who finished in less than half the median survey completion length to ensure quality responses. Drop-offs (those who left the survey before completing), terminations (those who were screened out as ineligible, over quota or did not meet security standards), and poor quality responses (those with gibberish (e.g., “sdfasdfjk”), nonsense (e.g., “good good good”), or straight lining (selects the same option throughout the survey) were distinguished from “good” completes (those who completed the survey without being terminated for either a screener or quality check) which were used to calculate the completion rate. Completion rate represents the number of individuals who complete the survey over the number of individuals who enter the survey using the formula: good completes/((good completes + terminations + over quotas) − (poor quality)). Demographic information on panel participants is not made available as this is proprietary information held by the panel partners, but our survey collected basic demographics on respondents.

To assess generalizability, we compared demographic data (age, sex, race, and ethnicity) of our survey respondents and weighted responses of those who reported they live in homes with firearms using Washington state and Colorado Behavioral Risk Factor Surveillance System data from 2020. BRFSS uses random digit dialing to survey noninstitutionalized adults aged 18 years or older about health-related risk behaviors. These data are weighted to be representative of their respective states. We found no meaningful differences between our sampled population and the BRFSS population, so we did not use survey weighting when analyzing our sample. To test for differences between subgroups (state of residence, owners vs non-owners (defined as all people living in gun owning homes who are not the firearm owner)), we used two-sample t tests for continuous variables and Fisher’s exact tests for categorical variables. An alpha level of 0.05 was used for significance testing. All analyses were performed using R Statistical Software (version 4.0.5; R Foundation for Statistical Computing, Vienna, Austria).

## Results

### Generalizability & demographics

The completion rate was calculated to be 54%. A total of 2201 people entered the survey, and we closed the survey after sample size was reached at 1029 quality completes; there were 898 terminations, 102 over quota, 122 poor quality responses, and 172 drop-offs. The final analytic sample included 1022 individuals. Results were similar across all demographic categories for each state (Additional file 2: Table S1). There were also few differences from our survey seen between states (Additional file 2: Table S2). Among our survey respondents, the majority of respondents were white (88.1%) and non-Hispanic (85.0%); half were female (50.8%), and the most common age group was ages 35–44 (25.5%; Table 1).

**Table 1:** Demographics of survey respondents

Demographics variable *N* (%) for categorical variables; Mean (SD) for continuous variables	Overall (*N* = 1022)
Age	44.8 (16.4)
18–24	116 (11.4%)
25–34	196 (19.3%)
35–44	260 (25.5%)
45–54	148 (14.5%)
55–64	137 (13.5%)
65 +	161 (15.8%)
Gender	
Male	497 (48.6%)
Female	519 (50.8%)
Other	2 (0.2%)
Prefer not to say	4 (0.4%)
Race (select all that apply)	
American Indian or Alaska Native	34 (3.3%)
Asian	31 (3.0%)
Black or African American	58 (5.7%)
Native Hawaiian or Pacific Islander	10 (1.0%)
White	900 (88.1%)
Prefer not to answer	20 (2.0%)
Ethnicity	
Hispanic/Latino	126 (12.3%)
Not Hispanic/Latino	869 (85.0%)
Prefer not to answer	27 (2.6%)
Education	
Less than high school diploma	24 (2.3%)
High school diploma or equivalency (GED)	325 (31.8%)
Some College	23(2.2%)
Associate degree (junior college)	247 (24.2%)
Bachelor's degree	258 (25.2%)
Master's degree	107 (10.5%)
Doctorate or Professional (MD, JD, DDS, etc.)	25 (2.4%)
Other	13 (1.3%)
Household income	
Less than $20,000	87 (8.5%)
$20,000–$39,999	160 (15.7%)
$40,000–$59,999	198 (19.4%)
$60,000–$79,999	158 (15.5%)
$80,000–$99,999	139 (13.6%)
$100,000–$149,999	148 (14.5%)
$150,000 or more	97 (9.5%)
Prefer not to answer	35 (3.4%)
Total number people in household	3.0 (1.6)
Children (aged 0–10) in household	0.5 (0.9)
Children (aged 11–18) in household	0.4 (0.9)
Firearm circumstances in household	
I personally own at least one firearm	696 (68.1%)
I do not personally own a firearm but I live in a home with firearms	326 (31.9%)

### Firearm handling and storage practices

Among respondents, 68.1% (*n* = 696) personally owned at least 1 firearm and the remaining 31.9% (*n* = 326) indicated they lived in homes with firearms but did not own firearms (Table 1). About a fifth of respondents indicated they handled their firearms once a week (17.8%) or once a month (21.2%), while nearly half (45.0%) said they handled their firearms once a year or less (Table 2). Just over one quarter (27.3%) of respondents indicated they had stored a firearm away from their home/car/garage in the last five years. The most common place respondents stored a firearm away from the home was at a family member’s home (39.1%) followed by a self-storage facility (35.3%). Storage at a firearm retailer was significantly more likely to be reported by the firearm owner compared to non-owners (*p* = 0.012 Table 2). Of those who stored a firearm away from home/car/garage in the last five years, nearly half (44.8%) indicated they stored the firearms for travel out of town for an extended period and was significantly more likely to be reported by the firearm owner compared to non-owners (*p* = 0.003, Table 2).

**Table 2:** Firearm ownership, handling, and storage by ownership status

	Firearm owner (*N* = 696) (%)	Firearm non-owner* (*N* = 326) (%)	Overall (*N* = 1022) (%)	*p* value**
I handle the firearm(s):				< 0.001
At least once a week	24.9	2.8	17.8	
At least once a month	28.2	6.4	21.2	
Less than once a month but at least once a year	28.0	16.6	24.4	
Less than once a year, but I have handled the firearm(s)	15.8	31.0	20.6	
I have never handled the firearm(s)	2.2	39.6	14.1	
I prefer not to answer	1.0	3.7	1.9	
Household member(s) who own at least one firearm (select all that apply)				
Myself	87.4	0.0	59.5	< 0.001
Spouse or partner	29.3	61.0	39.4	< 0.001
Another family member	6.9	31.3	14.7	< 0.001
Roommate/friend	3.6	8.3	5.1	0.002
Other	0.3	0.9	0.5	
Prefer not to answer	0.6	3.1	1.4	0.003
Has anyone in your household stored a firearm away from the home/car/garage in the last five years				< 0.001
Yes	32.0	17.2	27.3	
No	66.7	66.3	66.5	
Don't know	0.1	16.6	5.4	
Where were the firearms stored? (select all that apply)				
Friend or neighbor home	20.6	10.7	18.6	
Family member home	39.0	39.3	39.1	
Firearm retailer	22.0	7.1	19.0	0.012
Shooting range	16.6	14.3	16.1	
Law enforcement agency	8.1	5.4	7.5	
Military police or armory	4.0	0.0	3.2	
Pawn shop	6.7	1.8	5.7	
Self-storage facility	38.1	25.0	35.5	
Other	5.8	10.7	6.8	
Don't know	0.0	8.9	1.8	< 0.001
What were the circumstances? (select all that apply)				
Travel out of town for an extended period	49.3	26.8	44.8	0.003
Buying, selling or renting home	20.6	12.5	19.0	
Having young children in the home	22.9	17.9	21.9	
Having teenagers in the home	13.0	7.1	11.8	
Having older adults with dementia or other memory problems in the home	9.0	1.8	7.5	
Having someone with concerning mental health or substance use in the home	12.1	10.7	11.8	
Individual who is prohibited from having access to firearms is living or staying in the home	9.4	7.1	9.0	
Divorce or separation	4.9	0.0	3.9	
Military deployment	3.6	8.9	4.7	
For a relative who passed away	4.9	8.9	5.7	
During substance use, medical or mental health treatment of a household member	5.4	1.8	4.7	
Court order	3.1	1.8	2.9	
Other	5.4	14.3	7.2	
Prefer not to answer	3.1	7.1	3.9	

*Defined as all people living in gun owning homes who are not the firearm owner

**Only *p*-values less than 0.05 are presented

**Fig. 1:**
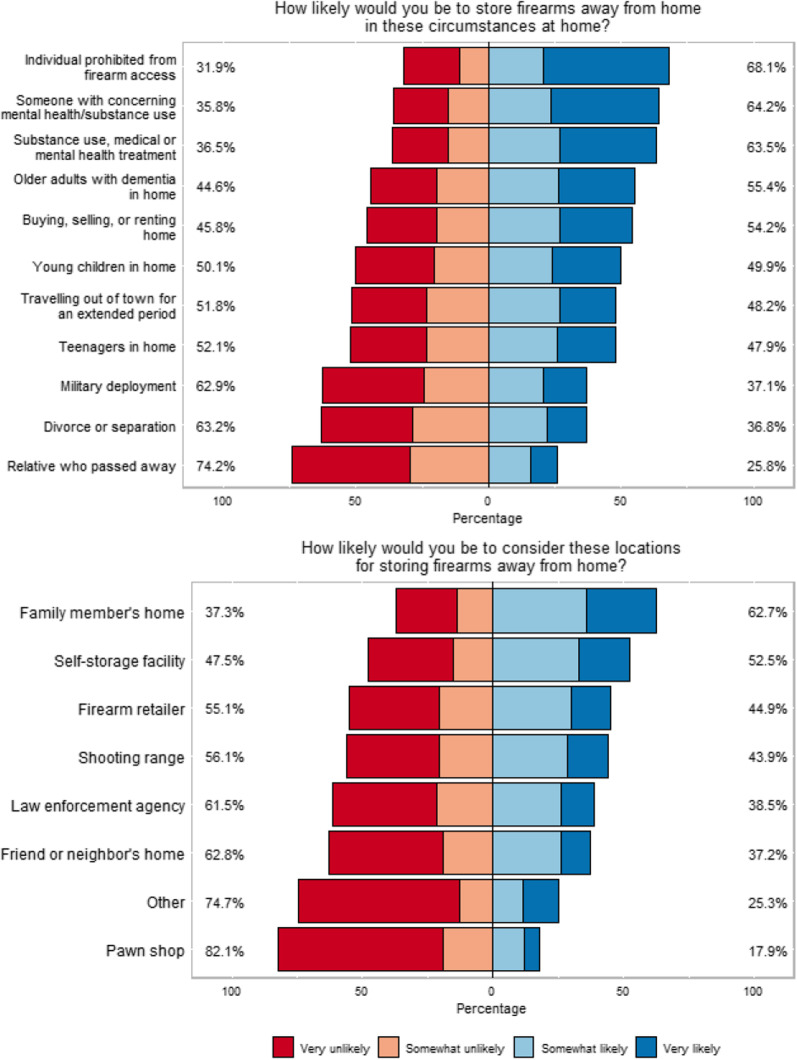
Circumstances precipitating storage and location of storage

The location where most respondents said they were somewhat or very likely to consider storing firearms away from the home was at a family member’s home (62.7%) or at a self-storage facility (52.5%). The only location with divergent views from firearm owners compared to non-owners was law enforcement agencies; 35.0% of those who own a firearm were somewhat or very likely to consider a law enforcement agency compared to 46.3% of non-owners (*p* = 0.007). A majority of respondents indicated that they would be somewhat or very likely to store away from the home or to encourage the firearm owner to store firearms away from your home in the following circumstances: having an individual who is prohibited from having access to firearms is living or staying in the home (68.1%); having someone with concerning mental health or substance use in the home (64.2%); having someone with substance use, medical or mental health treatment in the home (63.5%); having older adults with dementia in the home (55.4%); buying, selling, or renting the home (54.2%; Fig. 1).

### Map knowledge & acceptability

One-fifth (19.0%) of all respondents, and 24.4% of firearm owners, had heard of the gun storage map in their state. Most respondents (74.8%) said they would view it as positive if they learned a gun retailer or shooting range in their community was listed on the map and 67.3% would view it as positive if they learned a law enforcement agency was listed on the map. When asked “What would be the 3 best ways to share information in your community about options for voluntary, temporary firearm storage away from the home?”, survey respondents indicated that the best ways would be information provided at point-of-sale for firearms (57.9%), internet (55.6%), social media (44.5%), TV (35.8%), and sharing information via health care and mental health providers (30.1%). Sharing information via health care and mental health providers differed significantly by firearm ownership with 39.9% of non-owners indicating it was the best ways to share information compared to only 25.6% of firearm owners (*p* < 0.001; Table 3).

**Table 3:** Awareness of and outreach for Firearm storage maps

	Firearm owner (*N* = 696) (%)	Firearm non-owner* (*N* = 326) (%)	Overall (*N* = 1022) (%)	*p* value**
Have you heard of the Colorado/Washington gun storage map?				< 0.001
Yes	24.4	7.4	19.0	
No	75.6	92.6	81.0	
Would you view it as positive or negative if you learned a gun retailer or shooting range in your community was listed on the map?				0.002
Positive	76.7	70.6	74.8	
Negative	5.7	3.1	4.9	
Neither	17.5	26.4	20.4	
Would you view it as positive or negative if you learned a law enforcement agency in your community was listed on the map?				
Positive	68.0	66.0	67.3	
Negative	11.5	9.5	10.9	
Neither	20.5	24.5	21.8	
What would be the best ways to share information in your community about options for voluntary, temporary firearm storage away from the home? (Please select the three best ways)				
Information posted in community locations	30.6	35.6	32.2	
Information provided at point-of-sale for firearms	59.2	55.2	57.9	
TV	36.1	35.3	35.8	
Radio	23.0	18.7	21.6	
Newspapers	21.4	18.1	20.4	
Internet	55.5	55.8	55.6	
Social media	43.5	46.6	44.5	
Sharing information via health care and mental health providers	25.6	39.9	30.1	< 0.001
Other	27 (3.9	3.4	3.7	

*Defined as all people living in gun owning homes who are not the firearm owner

**Only *p*-values less than 0.05 are presented

### Concerns & facilitators for out-of-home storage

**Fig. 2:**
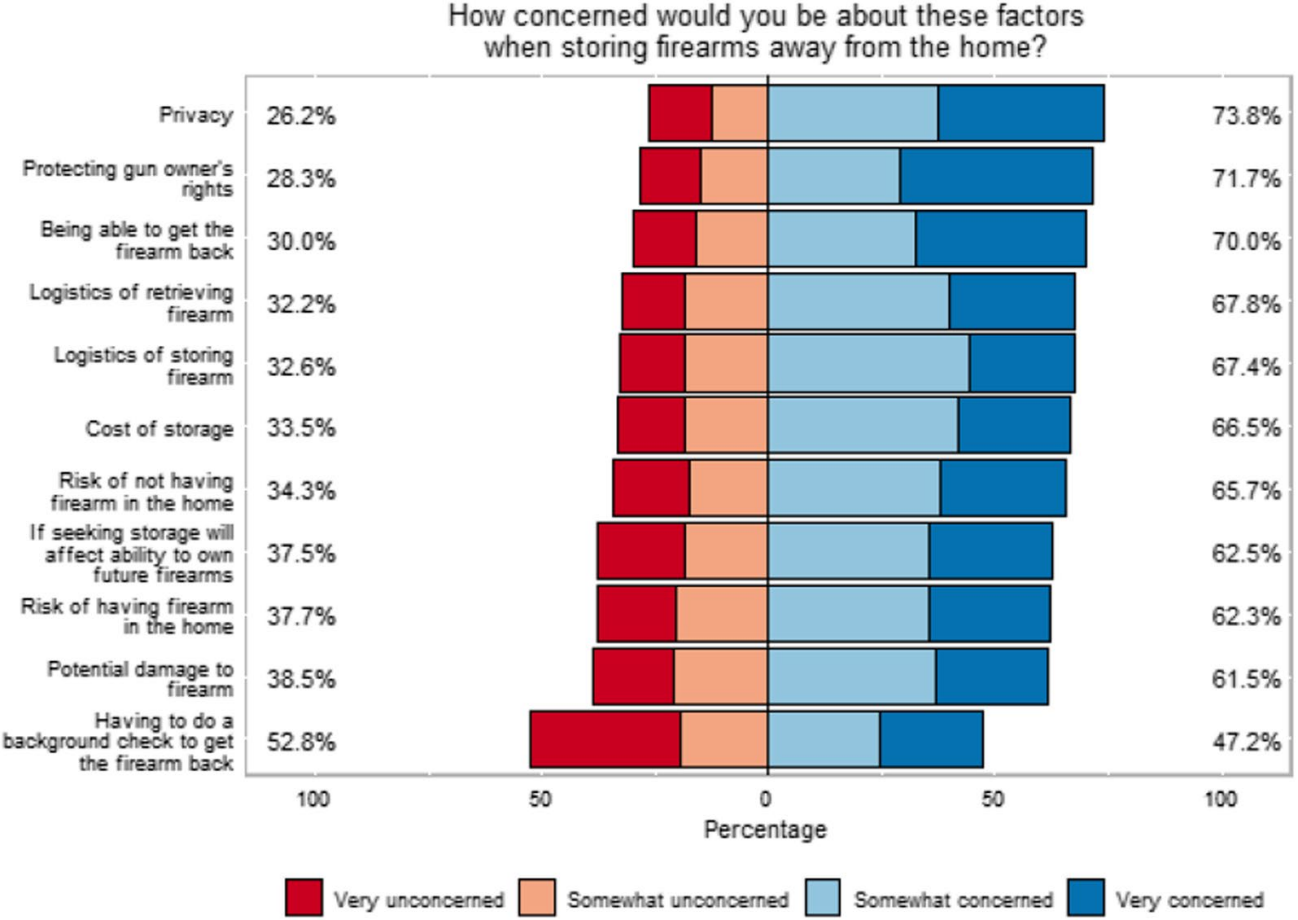
Concerns for out-of-home storage

When asked “How concerned would you be about each of these factors when storing or encouraging a household member to store firearms away from the home?”, 69% of people were somewhat or very concerned with being able to get the firearms back, 70.0% were concerned with privacy, and 71.5% were concerned with protecting the gun-owners’ rights (Fig. 2). For risk of *having* a firearm in the home, 69.1% of non-owners vs 59.3% of firearm owners indicated concern; for risk of *not* having a firearm in the home, 54.9% of non-owners vs 69.2% of firearm owners indicated concern (*p* = 0.013 and *p* < 0.001; respectively).

**Fig. 3:**
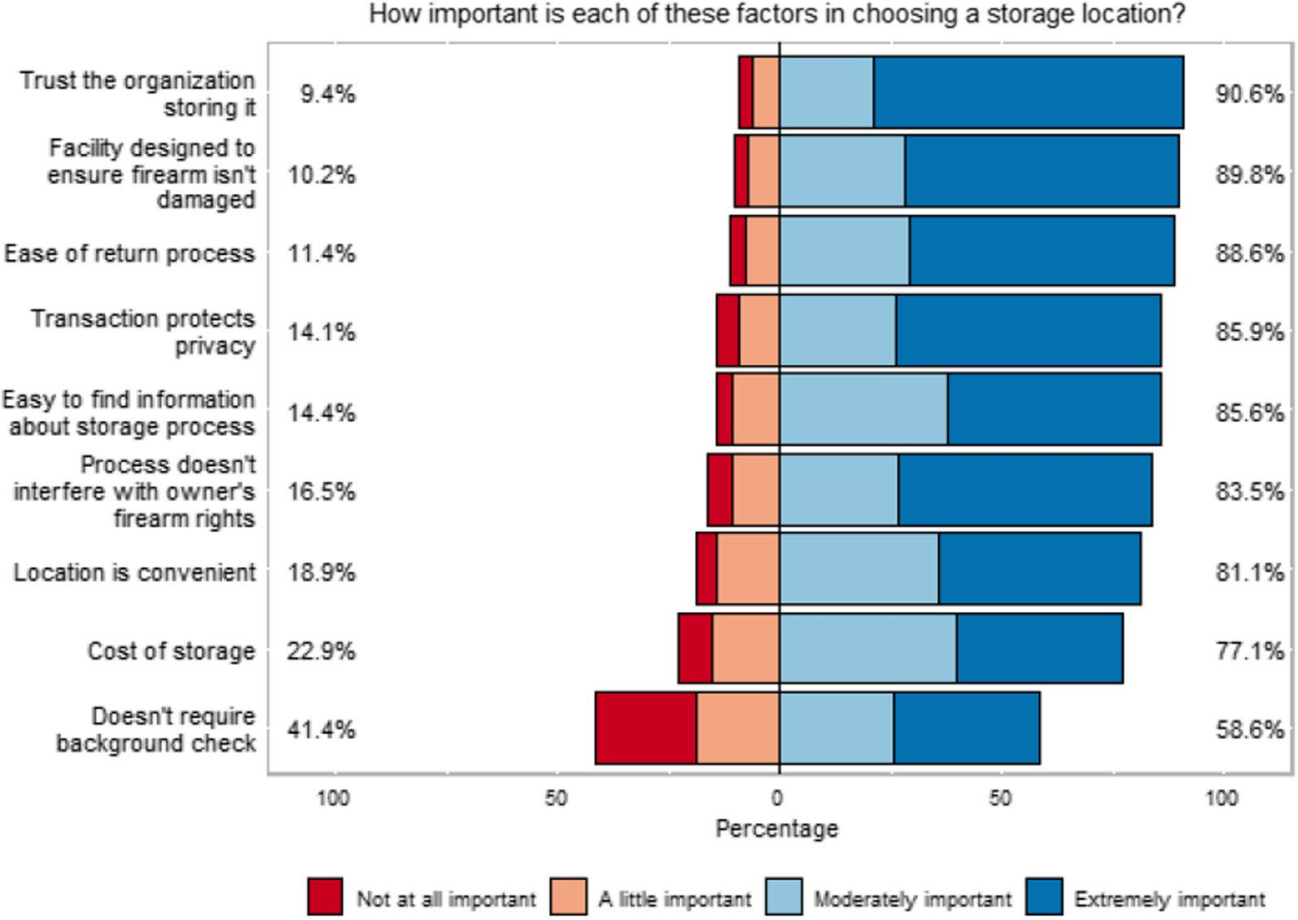
Important factors for storage location

In terms of choosing a potential storage location, the most important (moderately or extremely important) factors reported by survey respondents were trust in the organization storing the firearm (90.6%), facility designed to ensure the firearm is not damaged (89.8%), ease of the return process (88.6%), and that the transaction is privacy-protected (85.9%; Fig. 3). Privacy, protecting gun owner’s rights, whether seeking storage will affect ability to own firearms in future, potential damage to firearm, logistics of retrieving the firearm, and does not require a background check were all factors that were significantly more likely to be the moderately or extremely important to firearm owners compared to non-owners.

## Discussion

Nationally representative surveys indicate that approximately one-third of American adults report living in a home with a firearm (Parker et al. 2017). While safe storage (firearms locked, unloaded, or most desirably both) inside the home is something that a majority of firearm owners practice (Johnson et al. 2004; Berrigan et al. 2019) out-of-home storage practices, particularly among those who might store outside the home to prevent suicide or other firearm violence, need to be better understood. In this study, we found that 27.3% of people who either live in homes with firearms or who are firearm owners in Colorado and Washington state stored their firearm away from their home/car/garage in the last five years, most often at a family member’s home or at a self-storage facility. Our findings suggest consideration of out-of-home storage primarily in the situation of concerns for someone in the home and support for state maps.

Firearm storage maps, designed to connect firearm users with locations for out-of-home storage, should be publicized to those who have access to firearms. In this study, few respondents knew about the map, though there was overall support for the idea of the retailers/ranges and law enforcement agencies being listed on the map. Outreach and dissemination should be informed by this research indicating at point-of-sale for firearms, on the internet, social media, and TV were the ideal ways to share about options for storage away from the home. Gun Shop Projects (Polzer et al. 2020) are existing suicide prevention programs at point-of-sale in many states; outreach about out-of-home storage could be layered into these existing interventions. While suicide prevention should be included as a key reason for out-of-home storage and firearm maps, dissemination activities could also include other ways firearm owners currently or hypothetically use out-of-home storage options (e.g., extended travel, deployment, visitors in the home). This broad framing could normalize the idea of storage and also help destigmatize suicide risk.

Importantly, there were differences among those who own firearms compared to non-owners who live in homes with firearms, including sharing information via health care and mental health providers. This is consistent with previous studies that indicate some firearm owners may view interventions from healthcare or mental health providers as inappropriate due to their lack of knowledge of cultural or practical issues related to firearms (Knoepke et al. 2017; Shaughnessy et al. 1999). However, some lethal means counseling by physicians targeting specific populations such as parents of children or those presenting in the ED as suicidal may still be effective (Mueller et al. 2020; Runyan et al. 2016; Boggs et al. 2020). More research into the differences between firearm owners and those who live with firearms is needed. Even though non-owners may not (literally or legally) have a right to store the firearms they live with outside of the home, they still live with the potential risk of injury which accompanies proximity to them. Non-owners may differ from the primary firearm owner in terms of differences in beliefs and attitudes about out-of-home firearm storage. Enhanced understanding of household out-of-home storage decision making will help those who council for (physicians, mental health providers) lethal means safety among those with firearms in their homes.

Previous studies have described concerns of law enforcement agencies and retailers/ranges participating in out-of-home storage programs including logistical and liability concerns (Betz et al. 2022). Our study showed similar concerns of those with access to firearms about out-of-home storage programs. Understanding the motivations for firearm ownership (e.g., self-protection vs hunting) may help in counseling or developing tools to counsel firearm owners and non-owners on out-of-home storage options (Washington 2013; Butterworth et al. 2020). Seeking voluntary storage through a variety of businesses or organizations using firearm storage maps provides autonomy for the firearm owner and may help address the most important factors for storage location—trust in the organization storing the firearm. An additional recommendation for storage maps is a filter to find locations where the end user can coordinate with if they are in crisis. This might include an option to call 24/7 to drop off firearms outside of business hours or an option to search for large-scale storage in the case of multiple firearms. The proportion of people who would be willing to store a firearm outside of the home in the specific scenarios presented are relatively low. Concerns noted by those who live with firearms including cost, privacy, and process issues should be addressed to increase willingness to store firearms outside of the home. Expansion of and investment in firearm safety projects like Hold My Guns (2023) and the Gun Shop Project (2022) would likely both increase participation in storage maps and address cost as a concern from firearm owners. Additionally, recruitment of storage suppliers with various storage methods (such as with both storage where only store staff have access or rental storage lockers where the owner retains possession of the key) to participate in the map would be beneficial to ensure everyone seeking storage can find a process with which they are comfortable.

This study has several limitations. Non-probability quota samples do not allow a response rate to be calculated, and we do not have information on non-respondents. We addressed this limitation in several ways: (1) we were able to report a completion rate of 54%, indicating that over half of those who entered the survey completed it, and (2) comparing demographic data between our survey and the Behavioral Risk Factor Surveillance System Survey among the same study population (those who live in homes with firearms). Therefore, while screened-out bad quality responses (e.g., speeders, terminations, drop-offs, etc.) may reduce our findings’ generalizability to survey respondents who spent more time and effort, we can at least be reasonably assured that our survey is representative of our target population. The generalizability of these findings may still only be applicable to firearm-owning households in the two states sampled. It is possible the person who filled out the survey was misinformed about the practices of other household members in terms of out-of-home storage in the past or hypothetically in the future, and this may explain differences in out-of-home storage behavior and locations reported by firearm owners vs non-owners. However, only 5.4% of responses indicated they did not know about prior out-of-home storage suggesting the vast majority of our participants likely are aware of out-of-home storage practices and attitudes.

## Conclusion

Future work should further evaluate the differences in attitudes and beliefs of firearm owners versus non-owners who live in homes with firearms and examine out-of-home firearm storage specifically in a time of suicidality/mental health crisis. Navigating the details of ownership and autonomy over one’s own safety is complex when generally one person is legally considered the owner of a firearm, therefore limiting others’ autonomy over household firearm removal. This may help to explain differences in out-of-home storage behavior and preferred storage locations reported by firearms owners vs non-owners who live in homes with firearms. Additionally, in recognition that many firearm owners prefer storing with family members or friends, it is important to address legal obstacles to these types of temporary transfers during periods of acute risk (McCourt et al. 2017). Additionally, risk transferal to another household in the case of temporary storage with friends or family is also a concern; such storage could put members of that household at risk of harm. Out-of-home firearm storage is a relatively common practice and endorsed by many gun-owners in various circumstances, suggesting that storage programs are feasible and may be an acceptable approach to suicide prevention.

## Supplementary Information

**Additional file 1**. Survey Questions.

**Additional file 2**: **Table S1**. BRFSS survey result comparison. **Table S2**. Between state comparisons.

## Data Availability

The datasets used and/or analyzed during the current study are available from the corresponding author on reasonable request.
