# Supplementary material for: Practices, knowledge, and concerns for out-of-home firearm storage among those with access to firearms: results from a survey in two states
Source: Inj Epidemiol. 2023 Mar 13;10:15. doi: 10.1186/s40621-023-00426-9 (PMC10012481; doi:10.1186/s40621-023-00426-9)
Supplement: Supplementary file 1 — Additional file 1. Survey Questions. [file 40621_2023_426_MOESM1_ESM.docx]

**Aim 2b. User survey – 9/29/21**

| Screening questions | Are you at least 18 years old?   - Yes (eligible) - No (if no, exit survey)   Please enter your zip code:_______ (exclude if not WA or CO)  Do you live in a home in which a working firearm is kept in or around the home (whether or not you are the owner of the firearm)? By firearm, we refer to pistols, revolvers, shotguns and rifles. Firearms do not include air guns, BB guns, starter pistols or paintball guns. By working, we mean firearms that are in working order and capable of being fired.   - Yes - No (if no, exit survey) | |
| --- | --- | --- |
| Consent language | Thank you for being willing to share your perspective with us. This study is supported by a grant from the National Institutes of Health. Its purpose is to learn more about **temporary and voluntary** firearm storage which may reduce suicide**.** We want to learn about voluntary safety practices of gun-owners and their families. **This study is not about firearm policy.**  Your decision to complete this survey is completely voluntary. By completing it, you are consenting to participate and agreeing for your responses to be used for research purposes. You may stop completing the survey at any point, but if you wish to receive the participation incentive, you must complete all questions.  The research team will not have access to your name or any information that could be used to identify you. Your answers will be kept confidential. The research team will store survey responses on a secure server.  If you have questions about your rights as a participant you may make an anonymous inquiry at (303) 724-1055 or contact Dr. Betz, the Principal Investigator, at 720-724-2512. The study number is protocol #20-3042. | |
| Demographics/background information | What is your age?  Enter number: _______   - Prefer not to answer   Are you:   - Male - Female - Other (specify ___________) - Prefer not to answer   Race (choose all that apply):   - American Indian or Alaska Native - Asian - Black or African American - Native Hawaiian or Pacific Islander - White - Prefer not to answer   Ethnicity:   - Hispanic/Latino - Not Hispanic/Latino - Prefer not to answer   What is the highest degree you earned?   - Less than high school diploma - High school diploma or equivalency (GED) - Associate degree (junior college) - Bachelor's degree - Master's degree - Doctorate or Professional (MD, JD, DDS, etc.) - Other: _______( specify)   What is your approximate annual household income?   - Less than $20,000 - $20,000 to $39,999 - $40,000 to $59,999 - $60,000 to $79,999 - $80,000 to $99,999 - $100,000 to $149,999 - $150,000 or more - Prefer not to answer   How many people live in your household (including yourself)?  ____ (enter number)  How many children aged 0-10 live in your household?  ____ (enter number)  How many youth aged 11-18 live in your household?  ____ (enter number) | |
|  | Now we would like to ask you about the firearms in your home to understand who interacts with them and how often.  Which best describes your living circumstances as it relates to firearms?   - I personally own at least one firearm *(=OWNER for branching below)* - I do not personally own a firearm but I live in a home with firearms *(=NON-OWNER for branching below)*   We would like to know how often you handle the firearm(s) in your home – by handle we mean touch, clean, transport, or shoot the firearm. Please indicate which of the following best matches your experiences with the firearm(s) in your home.  I handle the firearm(s):   - At least once a week - At least once a month - Less than once a month but at least once a year - Less than once a year, but I have handled the firearm(s) - I have never handled the firearm(s)   Among those living in your household, who owns at least one firearm? (select all that apply)   - Myself - My spouse or partner - Another family member (parent, child, aunt, uncle, cousin) - A roommate/friend - Other | |
|  | FIREARM OWNER | NON-OWNER |
| Experiences seeking storage, including the request frequency and reasons (voluntary or ERPO-related) | Have you or anyone in your household stored a firearm away from your home, car or garage in the last five years?   - Yes (continue to next 2 questions) - No   Where have you stored firearms? (select all that apply)   - Friend or neighbor home - Family member home - Firearm retailer - Shooting range - Law enforcement agency - Military police or armory - Pawn shop - Self-storage facility - Other_______________   What were the circumstances? (select all that apply)   - Travel out of town for an extended period - Buying, selling or renting home - Having young children in the home - Having teenagers in the home - Having older adults with dementia or other memory problems in the home - Having someone with concerning mental health or substance use in the home - Individual who is prohibited from having access to firearms is living or staying in the home - Divorce or separation - Military deployment - For a relative who passed away - During substance use, medical or mental health treatment of a household member - Court order - Other (please specify) _________________ | To the best of your knowledge, has anyone in your household stored a firearm away from the home, car, or garage in the last five years?   - Yes (continue to next two questions) - No - Don’t know   Where were the firearms stored? (select all that apply)   - Friend or neighbor home - Family member home - Firearm retailer - Shooting range - Law enforcement agency - Military police or armory - Pawn shop - Self-storage facility - Other_________ - Don’t know   What were the circumstances? (select all that apply)   - Travel out of town for an extended period - Buying, selling or renting home - Having young children in the home - Having teenagers in the home - Having older adults with dementia or other memory problems in the home - Having someone with concerning mental health or substance use in the home - Individual who is prohibited from having access to firearms is living or staying in the home - Divorce or separation - Military deployment - For a relative who passed away - During substance use, medical or mental health treatment of a household member - Court order - Other (please specify) _________________ |
| Interest in seeking storage based on scenarios and locations | Next, we want to learn about when you think firearm storage outside of your home would be most useful, and where and how you would locate good storage options.  Imagine the following scenarios. How likely would you be to store firearms away from your home if someone in the household faced these circumstances? (Very likely, somewhat likely, somewhat unlikely, very unlikely)   - Travelling out of town for an extended period - Buying, selling or renting home - Having young children in the home - Having teenagers in the home - Having older adults with dementia or other memory problems in the home - Having someone with concerning mental health or substance use in the home - Individual who is prohibited from having access to firearms is living or staying in the home - Divorce or separation - Military deployment - Relative who passed away - During substance use, medical or mental health treatment of a household member   If you wanted to store firearms away from the home, how likely would you be to consider the following locations? (Very likely, somewhat likely, somewhat unlikely, very unlikely)   - Friend or neighbor’s home - Family member’s home - Firearm retailer - Shooting range - Law enforcement agency like police department or sheriff’s office - Pawn shop - Self-storage facility - Other____________________   If you wanted to store firearms away from the home for any reason, how would you find a storage location?   - Go to a place I already know provides storage - Internet searches - Ask friends or family for recommendations - Ask a health care provider - Ask firearm retailer or gun range - Ask law enforcement - Other (please specify) _________ - Not sure | Next, we want to learn about when you think firearm storage outside of your home would be most useful, and where and how you would locate good storage options.  Imagine the following scenarios. How likely would you be to encourage the firearm owner to store firearms away from your home if someone in the household faced these circumstances? (Very likely, somewhat likely, somewhat unlikely, very unlikely)   - Travelling out of town for an extended period - Buying, selling or renting home - Having young children in the home - Having teenagers in the home - Having older adults with dementia or other memory problems in the home - Having someone with concerning mental health or substance use in the home - Individual who is prohibited from having access to firearms is living or staying in the home - Divorce or separation - Military deployment - Relative who passed away - During substance use, medical or mental health treatment of a household member   If you were helping to find a place to store firearms away from the home, how likely would you be to consider the following locations? (Very likely, somewhat likely, somewhat unlikely, very unlikely)   - Friend or neighbor’s home - Family member’s home - Firearm retailer - Shooting range - Law enforcement agency like police department or sheriff’s office - Pawn shop - Self-storage facility - Other____________________   If you wanted to help find a place to store firearms away from the home for any reason, how would you find a storage location?   - Go to a place I already know provides storage - Internet searches - Ask friends or family for recommendations - Ask firearm retailer or gun range - Ask law enforcement - Ask a health care provider - Other (please specify) _________ - Not sure |
| Awareness of the gun storage map in their state and perceptions of participating organizations | Have you heard of the Colorado/Washington gun storage map?   - Yes - No   The gun storage map is designed to provide information for people seeking voluntary temporary firearm storage options to prevent suicide or for other reasons. Gun retailers, shooting ranges and law enforcement agencies throughout both states have agreed to be listed on the map.  Would you view it as positive or negative if you learned a gun retailer or shooting range in your community was listed on the map?   - Positive - Negative - Neither   Would you view it as positive or negative if you learned a law enforcement agency in your community was listed on the map?   - Positive - Negative - Neither | |
|  | OWNER | NON-OWNER |
| Barriers and facilitators to seeking storage | Imagine someone in your household was experiencing a mental health crisis and you were deciding whether to store firearms away from the home until the crisis passed. How concerned would you be about each of these factors? (Very concerned, somewhat concerned, somewhat unconcerned, very unconcerned) *randomize response order*   - Risk of having a firearm in the home - Risk of not having a firearm in the home - Being able to get the firearm back - Cost of storage - Privacy - Protecting my rights - Whether seeking storage will affect my ability to own firearms in the future - Potential damage to firearm - Logistics of storing firearm - Logistics of retrieving firearm - Having to do a background check to get the firearm back   How important would each of these factors be in choosing a storage location? (Very important, somewhat important, somewhat unimportant, very unimportant) – *randomize response order*   - Trust the organization storing it - Cost of storage - Location is convenient - Storage processes that do not interfere with my right to possess the firearm - Transaction handled in a way that protects my privacy - Easy to find information about storage process - Storage facility is designed to ensure firearm isn’t damaged - Ease of return process - Ability to store firearm without requiring a background check | Imagine someone in your household was experiencing a mental health crisis and you were deciding whether to encourage storage of firearms away from the home until the crisis passed. How concerned would you be about each of these factors? (Very concerned, somewhat concerned, somewhat unconcerned, very unconcerned) *randomize response order*   - Risk of having a firearm in the home - Risk of not having a firearm in the home - Being able to get the firearm back - Conflict with the firearm owner in my household if I encourage out-of-home storage - Whether a storage location will allow me to store since I’m not the owner - Cost of storage - Privacy - Protecting the owner’s rights - Whether seeking storage will affect their ability to own firearms in the future - Potential damage to firearm - Logistics of storing firearm - Logistics of retrieving firearm - Having to do a background check to get the firearm back   How important would each of these factors be in choosing a storage location? (Very important, somewhat important, somewhat unimportant, very unimportant) *randomize response order*   - Trust the organization storing it - Cost of storage - Location is convenient - Storage processes that do not interfere with my right to possess the firearm - Transaction handled in a way that protects our privacy - Easy to find information about storage process - Storage facility is designed to ensure firearm isn’t damaged - Ease of return process - Ability to store firearm without requiring a background check |
| Optimal avenues for public education on out-of-home storage | What would be the best ways to share information in your community about options for voluntary, temporary firearm storage away from the home? (select the three best)   - Information posted in community locations (flyers/brochures/billboards) - Information provided at point-of-sale for firearms - TV - Radio - Newspapers - Internet - Social media (eg Facebook, YouTube) - Sharing information via health care and mental health providers - Other (please describe):__________ | |
| Wrap-up | Please share any other comments or feedback you have about firearm storage here: | |
