# Supplementary material for: Practices, knowledge, and concerns for out-of-home firearm storage among those with access to firearms: results from a survey in two states
Source: Inj Epidemiol. 2023 Mar 13;10:15. doi: 10.1186/s40621-023-00426-9 (PMC10012481; doi:10.1186/s40621-023-00426-9)
Supplement: Supplementary file 2 — Additional file 2: Table S1. BRFSS survey result comparison. Table S2. Between state comparisons. [file 40621_2023_426_MOESM2_ESM.docx]

**Supplemental Tables**

Table 1: BRFSS Survey Result Comparison

|  | **Colorado** | | | **Washington** | | |
| --- | --- | --- | --- | --- | --- | --- |
|  | From 2020 BRFSS | From survey | 95% CI for survey* | From 2020 BRFSS | From survey | 95% CI for survey* |
| Age |  |  |  |  |  |  |
| 18-24 years | 12.1 | 11.8 | 8.2, 13.9 | 8.9 | 11.0 | 6.8, 12.2 |
| 25-34 years | 19.2 | 19.4 | 15.9, 23.2 | 13.9 | 19.0 | 16.2, 23.5 |
| 35-44 years | 17.1 | 27.6 | 24.3, 32.5 | 15.7 | 23.3 | 19.7, 27.5 |
| 45-54 years | 15.4 | 15.5 | 12.5, 19.1 | 16.1 | 13.7 | 10.3, 16.5 |
| 55-64 years | 18.1 | 12.5 | 9.8, 16.0 | 19.5 | 14.3 | 11.4, 17.9 |
| 65+ years | 18.0 | 12.1 | 10.4, 16.7 | 25.8 | 18.8 | 15.7, 23.1 |
| Race/Ethnicity |  |  |  |  |  |  |
| American Indian or Alaska Native, non-Hispanic** | - | 0.8 | 0.2, 2.1 | - | 1.2 | 0.5, 2.7 |
| Asian, non-Hispanic | 0.8 | 0.4 | 0.1, 1.5 | 4.1 | 3.7 | 2.2, 5.9 |
| Black, non-Hispanic | 1.5 | 5.1 | 3.2, 7.3 | - | 4.1 | 2.4, 6.1 |
| Hispanic | 10.4 | 13.3 | 9.8, 16.0 | 4.9 | 11.3 | 8.8, 14.7 |
| Multiracial, non-Hispanic | 1.5 | 1.6 | 0.7, 3.3 | 2.3 | 2.7 | 1.6, 4.9 |
| Native Hawaiian or Pacific Islander, non-Hispanic** | - | 0.2 | 0.0, 1.2 | - | 0.8 | 0.2, 2.1 |
| No race specified, non-Hispanic** | - | 0.6 | 0.1,1.8 | - | 0.4 | 0.0, 1.2 |
| White, non-Hispanic | 83.4 | 75.5 | 72.2, 80.0 | 82.3 | 73.2 | 69.0, 77.1 |
| Sex |  |  |  |  |  |  |
| Male | 56.7 | 49.4 | 46.2, 55.3 | 55.7 | 47.6 | 45.0, 54.2 |
| Female | 43.3 | 50.0 | 44.5, 53.6 | 44.3 | 51.8 | 45.4, 54.6 |
| *Confidence intervals are presented for percentages from this survey for each state and were calculated with the Clopper-Pearson method for a 95% confidence interval.  **Estimates with a total n<50 are suppressed. | | | | | |  |

Table 2: Between state comparisons

| **Demographics variable** | **Colorado**  (N = 510) | **Washington**  (N = 512) | **P value** |
| --- | --- | --- | --- |
| Age | 43.9 (15.8) | 45.7 (17.0) | 0.082 |
| 18-24 | 60 (11.8%) | 56 (11.0%) | 0.162 |
| 25-34 | 99 (19.4%) | 97 (19.1%) |  |
| 35-44 | 141 (27.6%) | 119 (23.4%) |  |
| 45-54 | 79 (15.5%) | 69 (13.6%) |  |
| 55-64 | 64 (12.5%) | 73 (14.4%) |  |
| 65+ | 67 (13.1%) | 94 (18.5%) |  |
| Gender |  |  | 0.416 |
| Male | 252 (49.4%) | 245 (47.9%) |  |
| Female | 255 (50.0%) | 264 (51.6%) |  |
| Other | 0 (0.0%) | 2 (0.4%) |  |
| Prefer not to say | 3 (0.6%) | 1 (0.2%) |  |
| Race (select all that apply) |  |  |  |
| American Indian or Alaska Native | 18 (3.5%) | 16 (3.1%) | 0.731 |
| Asian | 8 (1.6%) | 23 (4.5%) | 0.010 |
| Black or African American | 31 (6.1%) | 27 (5.3%) | 0.592 |
| Native Hawaiian or Pacific Islander | 4 (0.8%) | 6 (1.2%) | 0.753 |
| White | 449 (88.0%) | 451 (88.1%) | 1 |
| Prefer not to answer | 13 (2.5%) | 7 (1.4%) | 0.184 |
| Ethnicity |  |  | 0.630 |
| Hispanic/Latino | 68 (13.3%) | 58 (11.3%) |  |
| Not Hispanic/Latino | 429 (84.1%) | 440 (85.9%) |  |
| Prefer not to answer | 13 (2.5%) | 14 (2.7%) |  |
| Education |  |  | 0.458 |
| Less than high school diploma | 13 (2.5%) | 11 (2.1%) |  |
| High school diploma or equivalency (GED) | 163 (32.0%) | 162 (31.6%) |  |
| Associate degree (junior college) | 113 (22.2%) | 134 (26.2%) |  |
| Bachelor's degree | 133 (26.1%) | 125 (24.4%) |  |
| Master's degree | 57 (11.2%) | 50 (9.8%) |  |
| Doctorate or Professional (MD, JD, DDS, etc.) | 16 (3.1%) | 9 (1.8%) |  |
| Other | 15 (2.9%) | 21 (4.1%) |  |
| Household income |  |  | 0.341 |
| Less than $20,000 | 41 (8.0%) | 46 (9.0%) |  |
| $20,000 to $39,999 | 74 (14.5%) | 86 (16.8%) |  |
| $40,000 to $59,999 | 106 (20.8%) | 92 (18.0%) |  |
| $60,000 to $79,999 | 80 (15.7%) | 78 (15.2%) |  |
| $80,000 to $99,999 | 68 (13.3%) | 71 (13.9%) |  |
| $100,000 to $149,999 | 66 (12.9%) | 82 (16.0%) |  |
| $150,000 or more | 58 (11.4%) | 39 (7.6%) |  |
| Prefer not to answer | 17 (3.3%) | 18 (3.5%) |  |
| Total number people in household (mean (SD)) | 3.0 (1.6) | 3.0 (1.5) | 0.479 |
| Children (aged 0-10) in household (mean (SD)) | 0.4 (0.8) | 0.5 (1.0) | 0.455 |
| Children (aged 11-18) in household (mean (SD)) | 0.4 (0.8) | 0.4 (1.0) | 0.695 |
| Firearm circumstances in household |  |  | 0.947 |
| I personally own at least one firearm | 348 (68.2%) | 348 (68.0%) |  |
| I do not personally own a firearm but I live in a home with firearms | 162 (31.8%) | 164 (32.0%) |  |
